# Comparison of the treatment efficacy of herpes zoster neuralgia with temporary spinal cord stimulation at different sites

**DOI:** 10.3389/fneur.2025.1551164

**Published:** 2025-03-31

**Authors:** Xin Hu, Xiaolong Ye, Liu Wang, Yilun Wu, Zenghua Zhou, Shengrong Xu, Ruilin He, Zongbin Jiang

**Affiliations:** Department of Pain Medicine, The Second Affiliated Hospital of Guangxi Medical University, Nanning, China

**Keywords:** zoster-associated pain, temporary dorsal nerve root stimulation, temporary dorsal column stimulation, numerical rating scale, Pittsburgh Sleep Quality Index, QL-index scale

## Abstract

**Background:**

Zoster-associated pain (ZAP) is a common complication after herpes zoster infection. In recent years, conventional temporary dorsal column stimulation (tDCS) has been widely used nationally and internationally as a safe and effective minimally invasive treatment for ZAP. It has also been shown that temporary dorsal nerve root stimulation (tDNRS) may also be an effective treatment for ZAP. However, there is no direct clinical comparison between the newer tDNRS and the conventional tDCS.

**Objective:**

To compare the procedure time, radiation dose, efficacy and cost of the tDNRS and tDCS for the treatment of ZAP. And the complications of the two surgical modalities were recorded.

**Methods:**

Eighty patients with ZAP who attended the pain department of the Second Affiliated Hospital of Guangxi Medical University from January 2022 to July 2023 were selected. They were divided into tDNRS group (*n* = 40) and tDCS group (*n* = 40) by using random number table method. The operation time, radiation dose, number of electrodes used, cost of medical consumables, and number of postoperative electrical stimulation adjustments were recorded for each case, and the patients’ pain level, sleep quality, quality of life, and overall efficacy were analysed and compared at preoperative (T0), 1 week (T1), 1 month (T2), 2 months (T3) and 3 months (T4) after the operation.

**Results:**

A total of 76 patients were finally enrolled, 38 in the tDNRS group and 38 in the tDCS group. During the 3-month follow-up period, all patients showed a significant decrease in Numerical Rating Scale (NRS) and Pittsburgh Sleep Quality Index (PQSI) scores and a significant increase in quality of life (QL-Index scale) scores after treatment with both methods. And there was no statistically significant difference between the two methods. However, patients who received tDNRS had a significantly shorter operative time and less intraoperative radiation exposure than those who received tDCS (*p* < 0.0001), and the mean number of postoperative stimulation parameter adjustments and the cost of medical consumables were significantly lower than those in the tDCS group (*p* < 0.0001).

**Conclusion:**

Both tDNRS and tDCS were effective in the treatment of ZAP, but tDNRS had the advantages of more precise coverage, shorter procedure time, less radiation exposure, fewer electrical stimulation adjustments, and lower cost.

## 1 Introduction

Herpes zoster (HZ) is a viral dermatological condition resulting from the reactivation of the varicella-zoster virus (VZV) in individuals with impaired immune function (1). It primarily presents as unilateral clusters of herpes with severe neuralgia. Recent domestic and international epidemiological statistical surveys indicate that the annual incidence of HZ in the global population is approximately 3 to 5%. With the ageing of the population, this trend is observed to increase by 2.5 to 5.0% (2). Furthermore, the incidence of HZ in individuals over the age of 50 years is observed to rise significantly (3).

Zoster-associated pain (ZAP), including acute-phase pain and postherpetic neuralgia (PHN), is the most common and severe symptom and complication of HZ (4). Sansone highlighted that in addition to severe pain and sensory abnormalities, patients with ZAP are frequently accompanied by emotional and sleep impairments, with 45% of patients exhibiting moderate-to-severe affective disorders, including anxiety and depression. These symptoms have a significant impact on the quality of life of patients and their family members, leading to an increased financial burden on the family (5).

In recent years, temporary spinal cord stimulation (tSCS) has been widely used nationally and internationally as a safe and effective minimally invasive treatment for ZAP, with notable outcomes (6–8). With the development of neuromodulation technology, Jensen and Brownstone classified spinal cord stimulation (SCS) into the following three surgical procedures, based on the location of the electrodes placed in the epidural space of the spinal canal: traditional methods of spinal cord stimulation include dorsal column stimulation (DCS) (electrodes are placed in the epidural space, slightly lateral to the midline and towards the affected side), dorsal nerve root stimulation (DNRS) (electrodes are placed in the lateral epidural space, near the inner edge of the pedicle) and dorsal root ganglion stimulation (DRGS) (electrodes are placed in the intervertebral foramen) (9). DCS involves epidural electrode placement near the dorsal columns of the spinal cord, which modulates ascending pain signals by activating Aβ fibers and inhibiting nociceptive transmission via the gate-control theory. DNRS targets the lateral epidural space adjacent to the dorsal nerve root, enabling segmental inhibition of hyperexcitable nociceptive neurons at specific spinal levels, thereby providing precise coverage of radicular pain patterns. DRGS, in contrast, directly modulates the dorsal root ganglion (DRG), a critical site of neuropathic pain generation in ZAP, by stabilizing aberrant sodium channel activity and reducing ectopic discharges (10, 11). While tDCS remains widely used, its broad stimulation field may inadequately address focal dermatomal pain in ZAP. tDNRS was hypothesized to offer superior segmental specificity by targeting the dorsal root entry zone, where VZV-induced hyperexcitability predominates (12). At present, there are few reports on the efficacy of tSCS for the treatment of ZAP in these three different parts. This study compares only the two tSCS procedures, the temporary dorsal nerve root stimulation (tDNRS) and the temporary dorsal column stimulation (tDCS), and statistically analyses the operation time, radiation dose generated during the operation and the cost of medical consumables required for the operation of these two procedures. At the same time, indicators such as patients’ pre- and post-operative pain scores and quality of life are analysed to observe the efficacy, which is reported as follows.

## 2 Materials and methods

### 2.1 General characteristics of patients

This study was conducted in accordance with the ethical principles of the Declaration of Helsinki and approved by the ethics committee of the Second Affiliated Hospital of Guangxi Medical University (approval number: 2020KY-0128). Informed consent forms were signed by all patients and their families. According to the screening criteria of this study, patients with ZAP admitted to the pain department of our hospital from January 2022 to July 2023, regardless of gender, were recruited. According to the random number table method, they were randomly divided into the tDNRS group and tDCS group.

Inclusion criteria: (1) herpes lesions were all unilateral nerve involvement. (2) patients with ZAP (pain duration within 6 months after rash healing) and unsatisfactory pain control (NRS ≥ 4) after≥2 weeks of pharmacological therapy. (3) patients underwent temporary SCS therapy for a trial period of 7–14 days. (4) patients who had signed the informed consent form, were able to cooperate with follow-up questionnaires, speak clearly, and communicate easily.

Exclusion criteria: (1) ZAP patients with trigeminal nerve involvement. (2) patients treated with DRGS. (3) patients with insufficient electrical stimulation for less than 7 days or lost to follow-up due to electrode dislodgement, disconnection, or other reasons. (4) patients who received other interventional procedures during the 3-month follow-up period.

### 2.2 Apparatus and methods

#### 2.2.1 Main apparatus

The tSCS kit includes a stimulation electrode (Medtronic model 3,873, Medtronic, USA), an extension cable and electrical pulse generator (Medtronic model 355,531, Medtronic, USA), and a programmable controller (Medtronic model 8,840, Medtronic, USA).

#### 2.2.2 Surgical method

**Figure 1:**
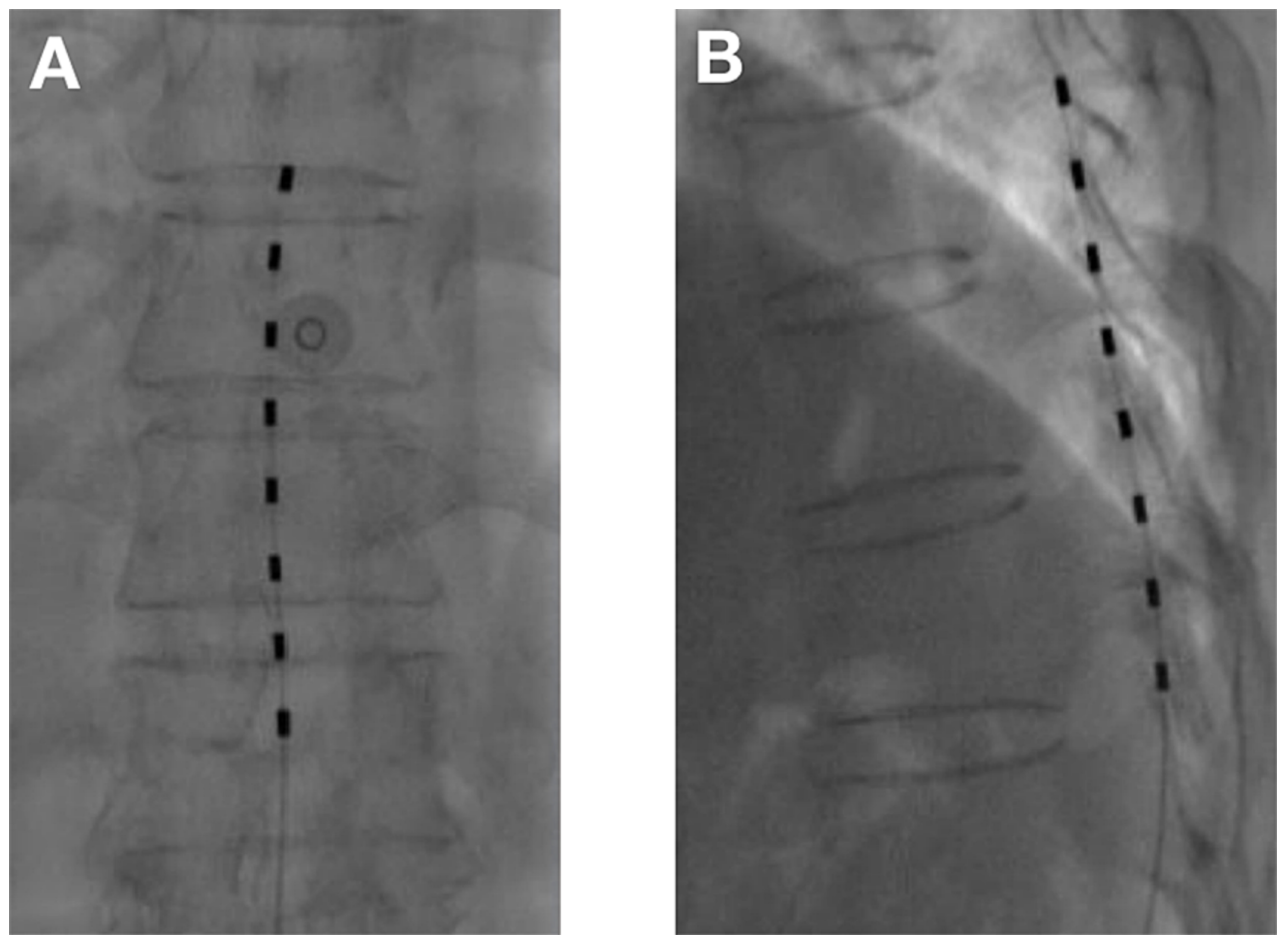
Temporary dorsal column stimulation with one electrode **(A)** Anterior–posterior view; **(B)** Lateral view.

**Figure 2:**
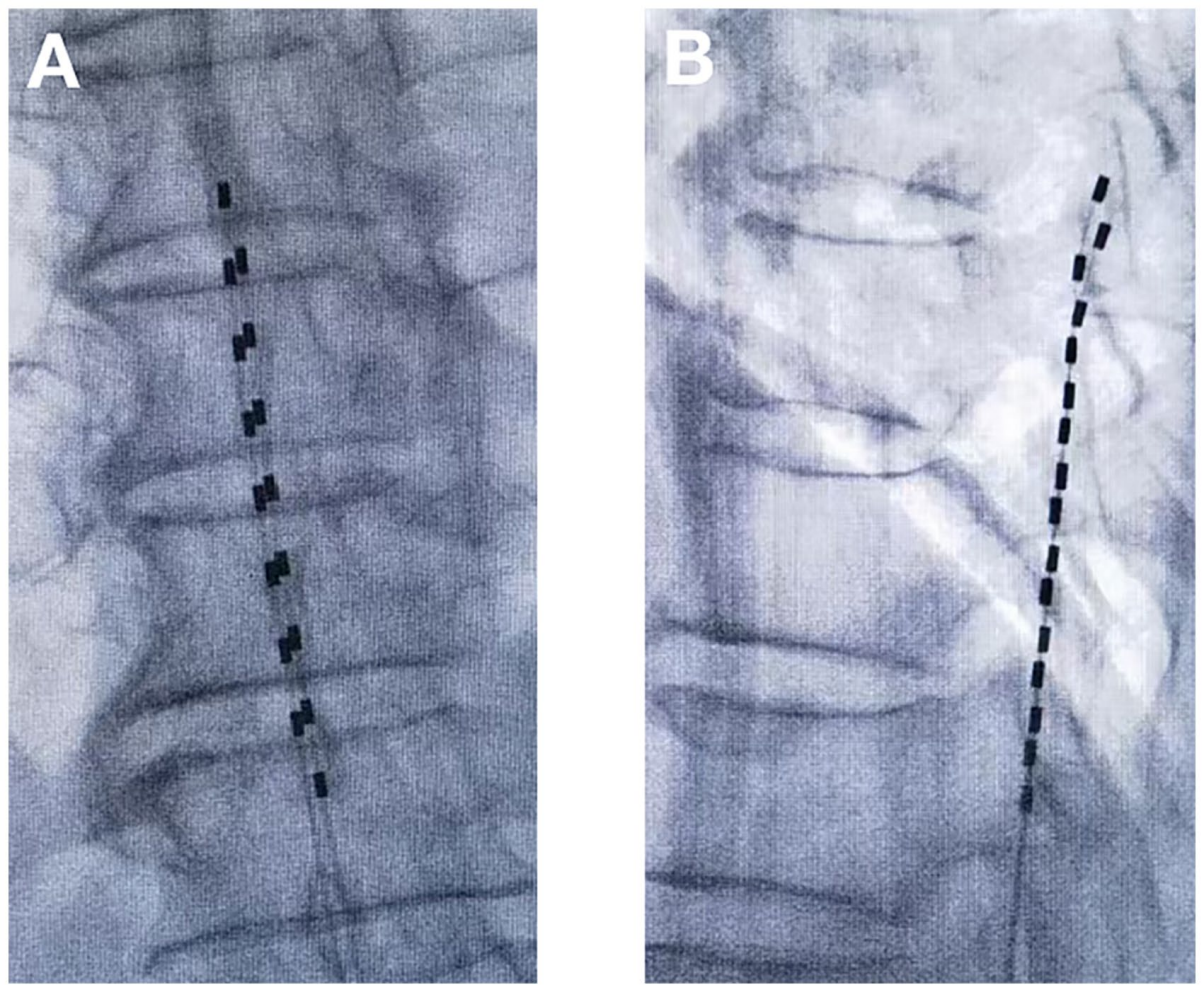
Temporary dorsal column stimulation with two electrodes **(A)** Anterior–posterior view; **(B)** Lateral view.

**Figure 3:**
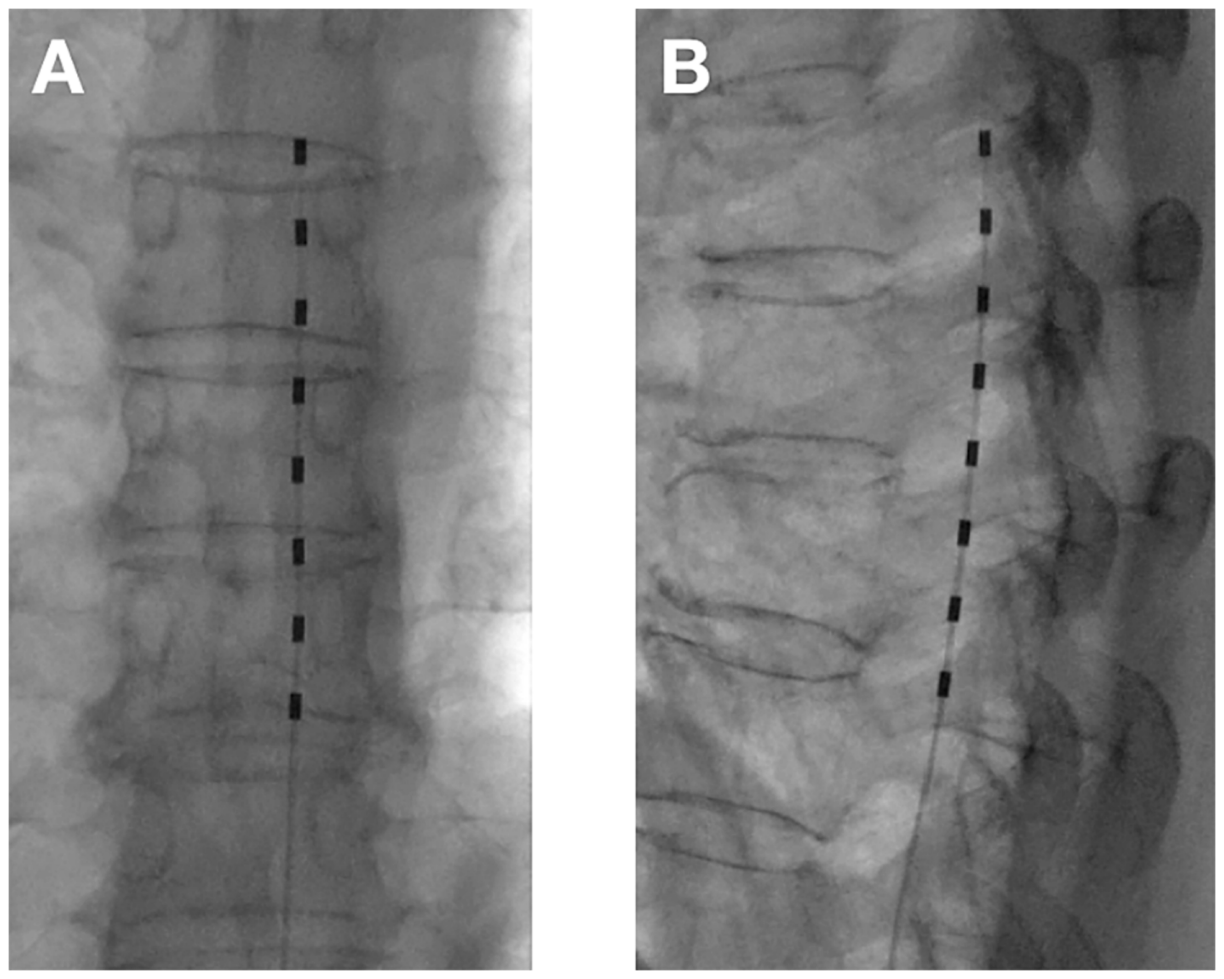
Temporary dorsal nerve root stimulation with one electrode **(A)** Anterior–posterior view; **(B)** Lateral view.

1. tDCS surgical procedure: The entire tDCS procedure was performed in the digital subtraction angiography (DSA) room. The patient was placed in the prone position. First, the spinous process interval was localized under the guidance of the DSA machine. After local anesthesia with 2 mL of 1% lidocaine, an epidural puncture was performed with a Tuohy needle. Under the guidance of the real-time DSA device, an 8-contact electrical stimulation electrode (Medtronic 3,873) was implanted into the epidural space. The implanted electrodes were placed in the midline of the target spinal segment, slightly offset to the affected side, and the test was performed. The position of the electrodes was slightly adjusted according to the test results until the stimulation paresthesia could cover the pain area. If one electrode could not provide the required coverage, a second electrode was implanted until the tingling sensation caused by electrical stimulation could cover more than 80% of the pain area (Figures 1, 2). If the test was successful, the puncture needle was withdrawn and the electrode lead was sutured to the skin. The patient would return to the ward after 15 min of observation with no discomfort.
2. tDNRS surgical procedure: The intraoperative operation of tDNRS was roughly the same as that of tDCS, except for the electrode position. tDNRS involved implanting an 8-contact test electrode into the lateral epidural space, and the middle contact position of the implanted electrode was placed on the inner edge of the pedicle of the affected nerve root (Figure 3).
3. Record the operating time and radiation dose generated during each operation.

### 2.3 Program control mode and frequency

Low-frequency electrical stimulation mode was generally used, and the range of programmed parameters was: frequency 40–60 Hz, pulse width 120–360 μs, voltage/current intensity adjusted according to the patient’s pain response. The specific programmed parameters were appropriate when the patient did not experience significant discomfort. The patient’s course of electrostimulation generally lasted between 7 and 14 days. Patients were given the same amount of analgesic medication during treatment. The number of electrical stimulation adjustments was based on the need for patients after surgery to cover the painful area as much as possible, and this could only be achieved by adjusting parameters such as contact points, pulse width or frequency. If only the intensity of the current or voltage was increased or decreased, it was not counted. The number of electrical stimulation adjustments was summed.

### 2.4 Observation indicators and assessment of efficacy

1. Numerical rating scales (NRS) were used to assess the pain level of the patients at preoperative (T0), 1 week (T1), 1 month (T2), 2 months (T3) and 3 months (T4) postoperatively. The scale was scored from 0 to 10, with mild pain scored from 1 to 3, moderate pain scored from 4 to 6, and severe pain scored from 7 to 10, with higher scores indicating more severe pain (13).
2. The Pittsburgh Sleep Quality Index (PSQI) was used to assess the sleep quality of the patients. The PSQI consists of 24 questions divided into 7 categories, with each category scoring between 0 and 3, with 0 indicating no sleep problems and 3 indicating extreme difficulty in sleeping, and the total score is obtained by adding up the scores of each category, and the total score ranges from 0 to 21, with higher scores indicating poorer sleep quality (14).
3. The QL-index scale was used to assess patients’ quality of life. It includes five aspects such as activity, health, support, daily life and general situation, with each item scored from 0 to 2. The higher the total score, the better the quality of life (15).
4. Efficacy was evaluated at T1, T2, T3, and T4. Decreases in NRS scores of >50% were considered effective. Overall efficacy rate = (number of cases with >50% reduction in NRS score/total number of cases × 100%) (16).
5. Complications, such as wound infection, cerebrospinal fluid (CSF) leak, epidural hematoma, nerve injury, electrode fracture, and electrode migration, were recorded during follow-up.

### 2.5 Statistical analysis

Statistical analyses were conducted using SPSS 25.0 (IBM Corp.). Continuous variables with normal distribution (verified by Shapiro–Wilk test) were expressed as mean ± standard deviation (SD), while categorical variables were presented as frequencies and percentages. Intergroup comparisons for normally distributed data were analyzed using independent samples *t*-test (with Levene’s test for homogeneity of variance), whereas non-normally distributed data were assessed by Mann–Whitney U test. Categorical variables were compared using Pearson’s χ^2^ test or Fisher’s exact test. Longitudinal changes in NRS, PSQI, and QL-index scores across timepoints (T0-T4) were evaluated through repeated measures ANOVA with Greenhouse–Geisser correction when sphericity assumption was violated, followed by Bonferroni-adjusted pairwise comparisons. Effect sizes were quantified using Cohen’s d (for t-tests) and partial η^2^ (for ANOVA). *p* < 0.05 was considered a statistically significant difference.

## 3 Results

### 3.1 Comparison of general information of patients in two groups

**Figure 4:**
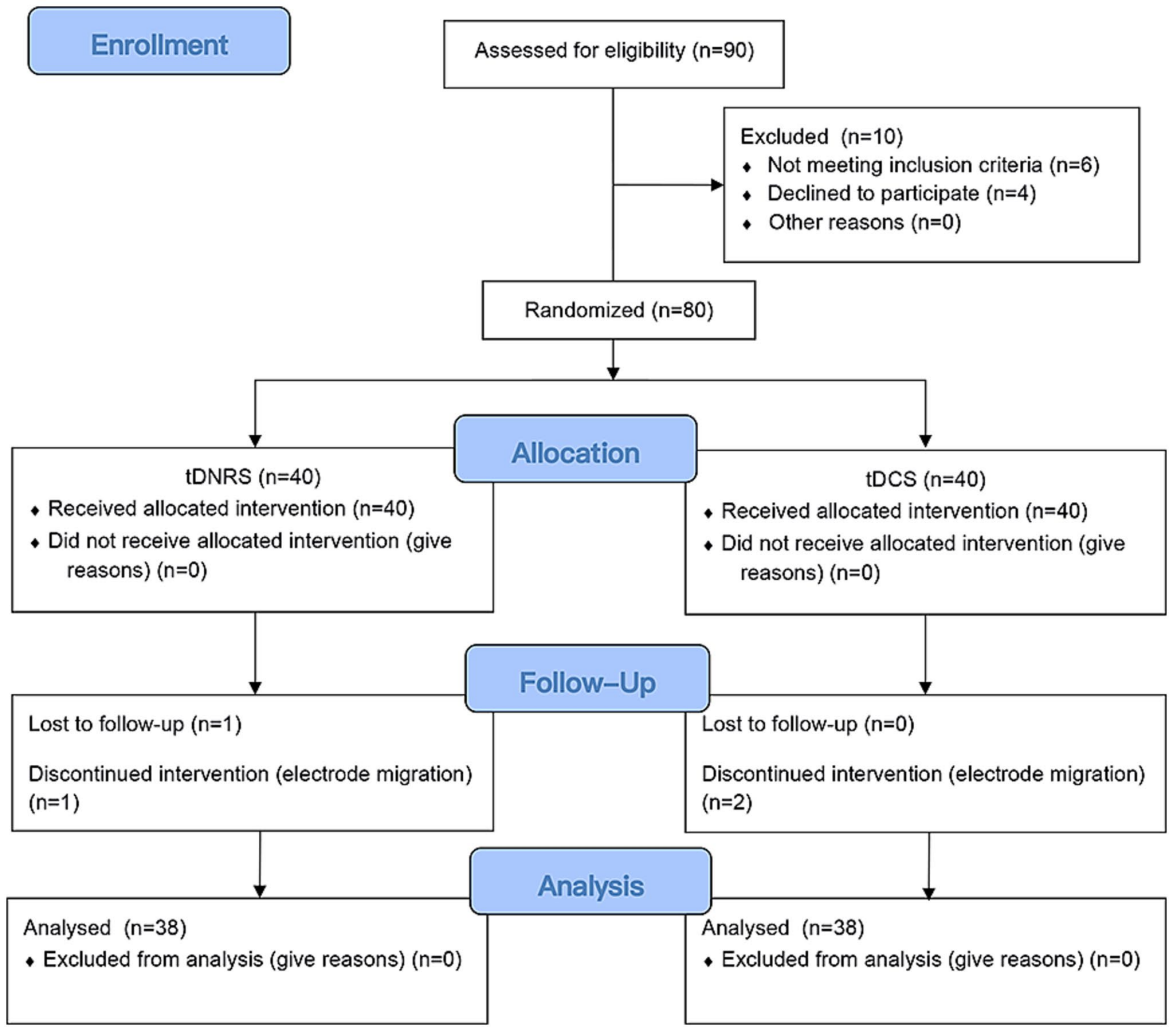
CONSORT flow diagram showing selection of study participants.

Initially, 90 patients were screened at the Second Affiliated Hospital of Guangxi Medical University. Among them, 10 individuals were excluded. 6 cases were excluded due to failure to meet inclusion criteria, and 4 cases declined to participate. Consequently, a total of 80 patients were enrolled in this study, 40 in the tDNRS group and 40 in the tDCS group. 1 patient in the tDNRS group was lost to follow-up and 1 patient withdrew from the study due to electrode migration. 2 patients in the tDCS group withdrew from the study due to electrode migration. Consequently, the final analysis included 76 patients, consisting of 35 males and 41 females within the cohort (Figure 4). There was no statistical difference in gender, age, disease duration, treatment segment, NRS score, PSQI score and QL-index score between the two groups preoperatively (*p* > 0.05, Table 1). All of them received a 3-month follow-up after surgery.

**Table 1:** Comparison of basic information in two groups.

Characteristic	Group		*P* value	Statistic
	tDCS (*n* = 38)	tDNRS (*n* = 38)		
Age (years)	64.2 ± 8.7	65.8 ± 9.2	0.439	0.779
Duration (months)	3.1 ± 0.6	3.3 ± 0.8	0.222	1.233
Gender (male/female)	18/20	17/21	0.818	0.053
Involved nerve			0.587	1.064
Cervical nerve	11	12		
Thoracic nerve	24	25		
Lumbar nerve	3	1		
NRS score	6.9 ± 0.8	7.1 ± 1.1	0.368	0.906
PSQI score	16.2 ± 1.4	16.8 ± 1.9	0.121	1.567
QL-Index scale	4.1 ± 0.7	3.9 ± 0.6	0.185	1.337

NRS: Numerical Rating Scale; PSQI: Pittsburgh Sleep Quality Index; tDCS: temporary dorsal column stimulation; tDNRS: temporary dorsal nerve root stimulation.

### 3.2 Comparison of NRS scores before and after treatment between the two groups

**Figure 5:**
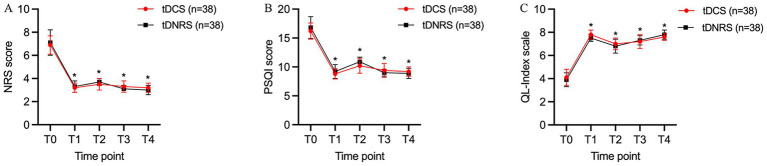
Improvement in pain levels, sleep quality and quality of life at post-operative time points in both groups. **(A)** Comparison of Numerical Rating Scale (NRS) scores in two groups; **(B)** Comparison of Pittsburgh Sleep Quality Index (PQSI) scores in two groups; **(C)** Comparison of QL-Index scores in two groups. In general, the changes of all three evaluated items for the two groups were almost the same, and there was no significant difference between the two groups at each time point. ^*^p < 0.05, compared with T0. (T0: preoperative; T1: 1 week postoperative; T2: 1 month postoperative; T3: 2 months postoperative; T4: 3 months postoperative).

The NRS scores of patients in both groups at each time point of T1, T2, T3, and T4 after treatment were significantly lower than those at T0, and the difference was statistically significant (*p* < 0.05). And the intergroup comparison showed that the difference in NRS scores between the two groups at each postoperative time point was not statistically significant (*p* > 0.05) (Figure 5A).

### 3.3 Comparison of PSQI scores before and after treatment between the two groups

The PSQI scores of patients in both groups at each time point of T1, T2, T3, and T4 were significantly lower compared with T0, and the differences were statistically significant (*p* < 0.05). There was no statistically significant difference in PSQI scores between the two groups compared with each time point after treatment (*p* > 0.05) (Figure 5B).

### 3.4 Comparison of QL-index scores before and after treatment between the two groups

Compared with T0, the QL-index scores of patients in both groups were higher at each time point of T1, T2, T3, and T4 after treatment, and the differences were statistically significant (*p* < 0.05). There was no significant difference in QL-index scores at the post-treatment time points when comparing between the two groups (*p* > 0.05) (Figure 5C).

### 3.5 Evaluation of the efficacy of the two groups

The overall efficacy of the two groups of patients at T1, T2, T3, and T4 was shown in Table 2. It could be seen that there was no statistically significant difference in comparing the overall efficacy rate between the two groups of patients at T1, T2, T3, and T4 after treatment (*p* > 0.05), which indicated that both surgical methods were effective in relieving pain.

**Table 2:** Comparison of effective rates (%) at each time point between the two groups.

Group	T1	T2	T3	T4
tDCS (*n* = 38)	74%	68%	71%	74%
tDNRS (*n* = 38)	76%	71%	79%	79%
*P* value	0.744	0.645	0.191	0.404
Statistic	0.107	0.212	1.707	0.695

tDCS: temporary dorsal column stimulation; tDNRS: temporary dorsal nerve root stimulation.

### 3.6 Comparison of the technical parameters of the two groups

Notably, the tDNRS technique not only produced similar therapeutic outcomes compared to tDCS, but also required lower overall cost, fewer post-operative stimulation adjustments, less radiation exposure, shorter procedural time, and fewer implanted electrodes than tDCS. Table 3 summarized the technical parameters of these two approaches.

**Table 3:** Comparison of technical parameters of the two groups.

Technical parameters	Group		*P* value	Statistic
	tDCS (*n* = 38)	tDNRS (*n* = 38)		
Number of electrodes used			0.007	7.370
One	29	37		
Two	9	1		
Procedure time (min)	54.2 ± 2.7	41.5 ± 2.1	< 0.0001	22.89
Radiation doses (mGy)	113.4 ± 14.5	72.3 ± 8.5	< 0.0001	15.07
Cost of medical consumables (yuan)	19,078 ± 536.2	16,342 ± 478.5	< 0.0001	23.47
Number of stimulation adjustments (time)	14.7 ± 1.2	8.4 ± 0.6	< 0.0001	28.95

tDCS: temporary dorsal column stimulation; tDNRS: temporary dorsal nerve root stimulation.

### 3.7 Comparison of complications between the two groups

During the course of the study, electrode migration occurred in 2 patients in the tDCS group and 1 patient in the tDNRS group, both of which had their electrodes removed in less than 1 week, and the patients had no obvious discomfort. The 76 patients finally included in the study showed no obvious complications and adverse reactions (Table 4).

**Table 4:** Comparison of complications between the two groups.

Complications	Group		*P* value	Statistic
	tDCS (*n* = 40)	tDNRS (*n* = 40)		
Electrode migration	2	1	0.556	0.346
Electrode fracture	0	0	-	-
Local wound infection	0	0	-	-
Nerve injury	0	0	-	-
CSF leak	0	0	-	-
Epidural hematoma	0	0	-	-

tDCS: temporary dorsal column stimulation; tDNRS: temporary dorsal nerve root stimulation; CSF: cerebrospinal fluid.

## 4 Discussion

Herpes zoster neuralgia is neuropathic pain that occurs in patients with HZ during outbreaks and after herpes has healed, which can have a long-term impact on the patient’s quality of life and place a burden on the socio-medical system (17), in addition to the fact that HZ is often seen in older adults with associated chronic diseases (18). Currently, conventional clinical treatments include drug therapy, nerve block and electrical stimulation therapy. The use of nerve block therapy in the clinic has some limitations because the elderly often have a combination of hypertension, diabetes or cardiovascular disease, and because the herpes area is sometimes extensive, often beyond a single ganglion. Firstly, nerve blocks require multiple injections at multiple sites (19), and secondly, nerve blocks also require glucocorticoid injections, which increase the risk of postoperative infection and blood glucose fluctuations, as well as the possibility of inadvertent drug entry into blood vessels, spinal cord injury and pneumothorax (20). Therefore, electrical stimulation therapy should be chosen as early as possible when conventional treatment fails. Electrical stimulation therapy is an established, safe, reversible and effective technique for relieving chronic neuropathic pain and improving patients’ function and quality of life (21, 22). Short duration electrical stimulation without pulse generator implantation is a safe and simple minimally invasive treatment for patients. tSCS is now being used in China to treat acute or subacute ZAP (23–25). After treatment with tSCS, patients experienced significant pain relief, greatly reducing the incidence of PHN.

Conventional SCS routinely places electrodes in the dorsal column of the spinal cord. However, the epidural space is wide in the middle and narrow on either side, making the electrodes easy to displace in the middle, and the electrical stimulation coverage is wide, making it difficult to accurately cover the pain caused by a single nerve lesion (26). The effectiveness of SCS for the treatment of ZAP had been reported in randomized controlled trials, systematic reviews and long-term retrospective studies (27–29). Levine et al. (30) reported the successful use of DNRS in the treatment of upper limb pain, interstitial cystitis (31) and diabetic neuropathy (32), demonstrating that SCS could be performed with electrodes placed on the dorsal root nerve in addition to the traditional electrode placement on the dorsal columns of the spinal cord. However, there are few reports in the literature on tDNRS.

In this study, short duration SCS was used, low pulse frequency mode was selected and patients with ZAP were divided into DNRS and DCS groups according to the randomized numerical table method and the efficacy and characteristics of these two procedures were compared. It was found that the NRS and PSQI scores of patients in both groups were significantly lower and the QL Index scores were higher at all postoperative time points than in the preoperative period, and the overall efficacy rates were greater than 50%. There was no statistically significant difference in the above indicators when comparing the two groups. This means that both surgical treatments are effective and tDNRS can relieve ZAP as well as tDCS and improve patients’ sleep quality and quality of life. A study comparing permanently implanted DNRS and SCS for the treatment of patients with chronic pain was also reported in 2017 by a Canadian research centre, which followed its patients for 12 months and found that the prognosis of patients with both procedures was excellent, ultimately concluding that DNRS could be used as an adjunctive therapy to SCS (29), which is similar to the conclusions of this study. Recent studies have demonstrated tDCS’s long-term analgesic efficacy (33), with 68% pain reduction sustained at 12 months in PHN patients (34). While Our 3-month tDNRS outcomes align with tDCS’s early-phase performance, longer-term data are needed to confirm durability.

However, we also observed inconsistencies between the two surgical approaches. Firstly, the proportion of the tDNRS group using a single electrode was greater than that of the tDCS group, suggesting that for the majority of patients treated with tDNRS, the use of a single electrode might be sufficient to cover the range of pain. This is because tDNRS can be more precisely localized to a single nerve (35), and for HZ patients with very definite radicular neuralgia (36), this type of electrical stimulation may be more likely to cover the area of pain, and as long as the electrodes are correctly positioned, the use of a single eight-contact electrode can provide complete coverage of all herpes and pain areas. While it is difficult to precisely cover a single nerve root lesion such as HZ with tDCS, it is often found that the electrical stimulation extends beyond the painful area, or that part of the painful area is not covered. The only way to avoid this is to add another electrode, i.e., to use the double electrode implantation method. For patients, tDNRS would significantly reduce their financial burden, as demonstrated by the results of this study. Our results further demonstrated that tDNRS required significantly shorter procedure time and fewer postoperative adjustments. This can be explained by anatomical differences in the epidural space. The midline epidural space (used for tDCS) is wider, allowing electrodes to shift easily and necessitating repeated fluoroscopic repositioning. In contrast, the lateral epidural space (used for tDNRS) is narrower, stabilizing the electrode near the nerve root and minimizing displacement (26). This inherent stability reduced intraoperative adjustments and postoperative parameter modifications, aligning with our data showing fewer stimulation adjustments and lower radiation exposure in the tDNRS group. Thus, DNRS can theoretically better compensate for the limitations of SCS, while maintaining the advantages of SCS and peripheral nerve stimulation (PNS) in terms of continuous stimulation and precise coverage of the painful area.

Although our study focused on the comparison of tDNRS with tDCS, emerging evidence suggests that DRGS also has favorable efficacy in the treatment of PHN (37). Both modalities target segmental pain pathways, but tDNRS achieves lateral epidural stimulation near the dorsal root entry zone, whereas DRGS requires precise electrode placement within the neural foramen to directly modulate the DRG. While DRGS demonstrates efficacy in case series (38, 39), it necessitates complex fluoroscopic navigation and carries risks of foraminal lead migration. Meanwhile, DRGS may benefit patients with focal radicular pain (e.g., single-level PHN), but tDNRS offers broader applicability for multi-dermatomal involvement (common in thoracic ZAP) through adjustable lateral electrode arrays.

## 5 Limitations

Limitations of this study: The sample size was relatively small, which limited the statistical analysis. The relatively short follow-up time could only demonstrate the short-term effects of tDNRS in the treatment of ZAP, and certain long-term effects (e.g., recurrence rate) were still uncertain. Later studies will increase the sample size and follow-up time to provide a stronger evidence-based rationale for treating ZAP with tDNRS. In addition to long-term follow-up, further studies on the efficacy and safety of repeated use of tSCS in patients with recurrent symptoms after initial successful treatment will be of interest.

## 6 Conclusion

Both tDCS and tDNRS have good efficacy in the treatment of ZAP, but tDNRS has the advantages of more precise coverage, shorter procedure time, less radiation exposure, fewer electrical stimulation adjustments, and lower cost.

## Data Availability

The original contributions presented in the study are included in the article/supplementary material, further inquiries can be directed to the corresponding authors.
